# Teriparatide mitigates oxidative stress following spinal cord injury and enhances neurological recovery via the Nrf2/HO-1 signaling pathway

**DOI:** 10.3389/fphar.2025.1538857

**Published:** 2025-03-19

**Authors:** Xiong Moliang, Ai Gangtong, Feng Yun, Luo Caiguang, Deng Liang, Guo Jia, Xiao Qiang

**Affiliations:** ^1^ Department of Orthopedics, Jiangxi Provincial People’s Hospital, The First Affiliated Hospital of Nanchang Medical College, Nanchang, China; ^2^ Department of Orthopedics, Shangrao People’s Hospital, The Shangrao Affiliated Hospital of Nanchang University, Shangrao, China

**Keywords:** spinal cord injury, Nrf2, teriparatide, oxidat ive stress, MDA

## Abstract

**Introduction:**

Spinal Cord Injury (SCI) represents a devastating form of central nervous system trauma, where oxidative stress plays a critical role in the ensuing pathology. Targeting oxidative stress presents a viable therapeutic avenue. Teriparatide, a synthetic analog of parathyroid hormone, is conventionally utilized for osteoporosis and bone defect management. Emerging evidence suggests teriparatide’s potential in modulating oxidative stress in ischemic stroke, yet its efficacy in SCI remains underexplored.

**Methods:**

We investigated the neuroprotective effects of teriparatide in a rat spinal cord injury (SCI) model. Teriparatide was administered to animals post-injury, and functional recovery was assessed using the open field test and Basso-Beattie-Bresnahan (BBB) locomotor rating scale. Molecular analyses included evaluation of Nrf2 pathway activation and antioxidant protein expression via immunofluorescence, Western blot, and ELISA. Additionally, glutathione peroxidase (GSH-PX) activity and malondialdehyde (MDA) levels were measured using commercial assay kits.

**Results:**

We obtained two significant results: Firstly, teriparatide treatment significantly enhanced motor function recovery post-SCI. Secondly, teriparatide upregulated Nrf2 expression, which subsequently increased the production of the antioxidant proteins HO-1 and SOD2, reduced MDA levels in spinal tissues, and boosted GSH-PX activity.

**Conclusion:**

Our findings demonstrate that teriparatide activates the Nrf2/HO-1 antioxidant pathway, effectively mitigating oxidative damage in SCI. This repositioning of an FDA-approved osteoporosis drug presents a clinically translatable strategy for neuroprotection.

## 1 Introduction

Spinal Cord Injury (SCI) represents a severe condition within the spectrum of spinal disorders, leading to significant impairments in motor skills, sensory functions, and autonomic control below the site of injury. It is a prominent cause of disability and mortality, particularly in middle-aged and older adults (Hu et al., 2023). Recent societal advancements have been accompanied by a surge in SCI incidence. The resulting complications and long-term effects not only pose a grave risk to individual health but also exert considerable strain on social and healthcare systems, highlighting its status as a critical public health concern (de Almeida et al., 2023; Zheng and Tuszynski, 2023). The pathophysiology of SCI is categorized into primary and secondary phases. The primary injury is the immediate consequence of mechanical trauma to the spinal cord. In contrast, secondary injury arises from a cascade of biological processes including oxidative stress, ferroptosis, and inflammatory responses that originate from the initial trauma and serve to amplify and propagate the damage (Sterner and Sterner, 2022; Shao et al., 2019). Given that primary injury is an immediate result of trauma and thus largely inevitable, research into the mechanisms underlying secondary injury holds significant therapeutic potential for improving SCI outcomes.

Following SCI, the affected region undergoes transient hypoxia due to hemorrhage and vasodilation. This hypoxia can precipitate oxidative stress through an inflammatory response that entails neuronal hyperactivity and glutamate excitotoxicity (Yu et al., 2023). Oxidative stress arises when the antioxidant defenses of the system are overwhelmed, leading to an overproduction of reactive oxygen species (ROS). These ROS can cause neuronal lipid peroxidation, DNA damage, and subsequent cell death (Yu et al., 2023). Antioxidant therapy has the potential to mitigate oxidative stress and thus enhance neural function recovery post-injury.

Methylprednisolone is the current drug of choice for acute SCI management due to its free radical scavenging and anti-inflammatory properties. Nonetheless, its clinical application must be judiciously considered in light of its numerous adverse effects, including gastric mucosal bleeding, increased risk of postoperative infections, pulmonary embolism, and the possibility of long-term avascular necrosis of the femoral head (Lambrechts et al., 2023). Furthermore, benzothiazole compounds, such as tizanidine, have been investigated for their potential neuroprotective effects, which are thought to arise from their ability to block ion transport proteins and diminish presynaptic glutamate release. However, there is a paucity of robust clinical evidence to support their efficacy (Lambrechts et al., 2023). Currently, effective pharmacological treatment methods for promoting functional recovery following SCI remain limited (Zuo et al., 2023). Consequently, the development of new antioxidant medications is imperative to enhance the prognosis for individuals with spinal cord injuries.

Teriparatide, a synthetic analogue of parathyroid hormone (PTH), is widely used in studies addressing bone defects, nonunion fractures, and related disorders (Yuan et al., 2019; Li et al., 2023; Berg et al., 2003). Recent investigations have revealed that PTH can modulate oxidative stress by scavenging reactive oxygen species in bone marrow mesenchymal stem cells, thus maintaining cellular viability and preventing apoptosis (Wang et al., 2023). Our initial findings indicate that teriparatide may mitigate oxidative stress in the middle cerebral artery occlusion (MCAO) model by enhancing the production of nuclear factor erythroid 2-related factor 2 (Nrf2) and stimulating the expression of the antioxidative enzymes heme oxygenase-1 (HO-1) and superoxide dismutase 2 (SOD2), which notably ameliorates symptoms of neuronal injury (Xiong et al., 2022). Despite its promise, research on the efficacy of teriparatide in counteracting oxidative stress following SCI remains scant both domestically and internationally. This study utilizes a rat SCI model treated with teriparatide to assess its potential in reducing oxidative stress and improving outcomes post-injury. Furthermore, we aim to elucidate the underlying mechanisms of teriparatide’s antioxidative effects.

## 2 Materials and methods

### 2.1 Experimental animals

For this study, we utilized 138 male Sprague-Dawley (SD) rats, aged 8 weeks and with body weights ranging from 240 to 300 g. These rats were supplied by Jiangxi Zhonghong Boyuan Biotechnology Co., Ltd. The Animal Experiment Center of the First Affiliated Hospital of Nanchang University provided specific pathogen-free housing conditions for the animals, which were overseen by trained personnel. Individual housing was provided for each rat, along with unrestricted access to water and standard laboratory chow. The facility maintained an ambient temperature of 22°C–24°C, a relative humidity around 60%, and a 12-h light-dark cycle to simulate natural conditions. The study protocol received approval from the Animal Ethics Committee of the First Affiliated Hospital of Nanchang University, ensuring adherence to ethical standards. All experimental procedures were performed in accordance with the National Institutes of Health’s Guide for the Care and Use of Laboratory Animals (NIH publication No. 8023, revised 1978), ensuring the welfare of the animals throughout the study.

### 2.2 Preparation of Rat SCI model

Rats were subjected to an 8-h period of fasting and water restriction before the surgical procedure. Anesthesia was initiated via an intraperitoneal injection of 1% pentobarbital sodium at a dose of 0.3 mL/100 g body weight. The rat SCI model was established employing Allen’s method. Following the confirmation of effective anesthesia, rats were positioned prone, and a surgical dissection was performed to expose the T10 spinal cord by removing the T10 lamina. A precision impactor applied a controlled blunt contusion to the T10 spinal cord with specified parameters: a depth of 1.5 mm, a velocity of 5 m/s, and a duration of 0.5 s. Indicators of a successful SCI model included immediate tail spasms, involuntary hind limb and torso movements, subsequent hind limb paralysis, and observable spinal cord swelling and hemorrhage. Inclusion criteria for the experiment post-modeling were as follows:1) dragging of the hind legs upon recovery from anesthesia, 2) complete motor function loss in both hind limbs, and 3) a Basso, Beattie, and Bresnahan (BBB) score ranging from 0 to 1. The sham-operated group underwent identical surgical procedures, excluding the spinal cord contusion. Postoperative care involved daily administration of 80,000 units of penicillin for 3 days to prevent infection and bi-daily manual bladder and bowel expression to facilitate elimination.

### 2.3 Administration of pharmaceuticals

At 3 h post-establishment of the SCI model, the administration of pharmaceuticals commenced. Teriparatide was administered at a dosage of 20 μg/kg/day via subcutaneous injection (Shanghai Macklin Biochemical Co., Ltd., Catalogue No. T860423) (Kendler et al., 2017), ML385 was administered at a dosage of 30 mg/kg/day via intraperitoneal injection (Shanghai Macklin Biochemical Co., Ltd., Catalogue No. M872746) (Shan et al., 2021), and a saline solution was administered at a dosage of 20 μg/kg/day via subcutaneous injection (Sichuan Karen Pharmaceutical Co., Ltd.). These administrations were conducted consistently at the same time each day. The administration period for teriparatide, ML385, and saline varied according to experimental requirements, with a maximum duration of 14 days.

### 2.4 Assessment of neurological function recovery through open field test and Basso, Beattie, and Bresnahan scoring

Neurological function recovery in rats was evaluated using open field tests administered on postoperative days 3, 7, and 14. Additionally, Basso, Beattie, and Bresnahan (BBB) scoring was conducted preoperatively and on postoperative days 1, 3, 7, and 14. Prior to the open field test, rats were acclimatized for 1 hour in a behavioral laboratory within an opaque black reaction box measuring 100 cm in base length and 40 cm in height. A camera positioned 100 cm above the box captured the rats’ movements, recording their speed and distance traveled over a 10-min duration. The open field apparatus was sanitized with alcohol before each session, and following each trial, fecal matter was meticulously removed. Each rat underwent testing in the apparatus only once. Subsequent to each trial, the reaction box was thoroughly cleaned to eliminate fecal matter. Movement trajectories and distances were quantified using EthoVision XT 15 software. Motor function was subsequently evaluated using the BBB scoring scale.

### 2.5 Immunofluorescent staining for the detection of Nrf2, HO-1, and SOD2 expression

Rats were successfully anesthetized by intraperitoneal injection of 1% sodium pentobarbital at a dose of 0.3 mL/100 g body weight and subjected to cardiac perfusion after 3, 7, and 14 days of treatment. Perfusion was performed with 200 mL of ice-cold saline, followed by 200 mL of 4% paraformaldehyde. Subsequently, the injured spinal cord segments were excised. The tissues were dehydrated using an automated tissue processor and then embedded in paraffin. Sections were cut at a thickness of 4 µm for immunohistochemical analysis. The immunohistochemistry procedure involved a two-step method. Sections were deparaffinized in xylene, rehydrated through graded alcohols, and subjected to antigen retrieval in citrate buffer (pH 6.0). Membrane permeabilization was achieved using 0.2% Triton X-100. Non-specific binding was blocked with 5% normal goat serum at room temperature. Sections were incubated overnight at 4°C with primary antibodies against Nrf2 (Sigma, 1:200, SAB4501984), HO-1 (Abcam, 1:200, ab189491), and SOD2 (Abcam, 1:200, Ab13534). This was followed by a 1-h incubation with a goat anti-rat 488 secondary antibody (Thermo Fisher, 1:1000, A48262) and subsequent mounting with an anti-fade mounting medium. Fluorescence microscopy, utilizing a Nikon upright microscope, was employed to examine the staining outcomes for each experimental group.

### 2.6 Quantification of Nrf2 expression in spinal cord tissues by ELISA

Following euthanized by intraperitoneal injection of an overdose of 1% pentobarbital sodiumat a dose of 200 mg/kg body weight, spinal cord tissues were extracted from the subjects in each experimental group. A specimen from the affected region was excised and transferred to an EP tube. This specimen was subsequently homogenized in PBS (pH 7.2–7.4) at a 1:9 tissue-to-buffer ratio with the aid of an ultrasonicator (Fisher Scientific, United States). Post-homogenization, the homogenate was centrifuged at 2,000–3,000 rpm for 20 min at 4°C, and the resulting supernatant was meticulously collected. The supernatant was then applied to a rat-specific Nrf2 ELISA kit (Jiangsu Enzyme Immunoassay Industry Co., Ltd.) to quantify Nrf2 levels across the different groups.

### 2.7 Western blot analysis of protein expression

In this study, rats from each experimental group were successfully anesthetized by intraperitoneal injection of 1% sodium pentobarbital at a dose of 0.3 mL/100 g body weight and subsequently perfused with 200 mL of physiological saline via cardiac infusion to prepare for spinal cord tissue collection. A section of the injured spinal cord tissue was excised and placed into an EP tube. The tissue was lysed using RIPA buffer (Solabao Technology Co., Ltd., Beijing) supplemented with PMSF protease inhibitor (Solabao Technology Co., Ltd., Beijing) to extract total protein. Protein concentrations were quantified using the BCA Protein Assay Kit (Beyotime). The samples were denatured by heating at 100°C for 5 min with Loading Buffer and then stored at −80°C. Proteins were separated by SDS-PAGE and electrotransferred onto a PVDF membrane. The membrane was blocked with 5% BSA for 1 h and subsequently incubated with primary antibodies targeting HO-1 (abcam, 1:2000, ab189491), SOD2 (abcam, 1:2000, Ab13534), and β-actin (Bioworld, 1:1000, AP0060) overnight at 4°C. Following primary antibody incubation, the membrane was washed with TBST and incubated with horseradish peroxidase-conjugated secondary antibodies (Zhongshan Golden Bridge, 1:5,000, ZB2301) for 1 h at room temperature. After additional washing with TBST, protein bands were visualized using chemiluminescent ECL detection reagent.

### 2.8 Assessment of glutathione peroxidase activity and malondialdehyde content in spinal cord tissues

Spinal cord samples were collected from rats in each experimental group following successful anesthesia, which was induced by an intraperitoneal injection of 1% pentobarbital sodium at a dose of 0.3 mL/100 g body weight. The samples were initially rinsed to eliminate any residual blood and carefully dried using filter paper. Following accurate weighing, the tissues were transferred into Eppendorf tubes and homogenized in a physiological saline solution to achieve a 10% (w/v) homogenate. This process was performed using an ultrasonicator (Fisher Scientific, United States) to ensure complete homogenization. The resultant mixture was then centrifuged at 3,000 rpm and 4°C for 15 min, after which the supernatant was meticulously collected. For the quantitative analysis of glutathione peroxidase (GSH-PX) activity and malondialdehyde (MDA) content, the supernatant was introduced into respective assay kits: a GSH-PX activity assay kit (Nanjing Jiancheng Bioengineering Institute, A005-1-2) and an MDA assay kit (Nanjing Jiancheng Bioengineering Institute, A003-1-2). The assays were conducted in strict accordance with the manufacturer’s protocols.

### 2.9 Hematoxylin and eosin staining to observe lesions and cell degeneration

After 3, 7, and 14 days of treatment, the experimental rats were anesthetized via intraperitoneal injection with 1% pentobarbital sodium at a dosage of 0.3 mL per 100 g of body weight. Subsequent to anesthesia, the rats were subjected to intracardiac perfusion with 200 mL of ice-cold saline, succeeded by an equivalent volume of 4% paraformaldehyde. Spinal cord tissues were excised, dehydrated using an automated tissue processor, embedded in paraffin, and sectioned into slices 4 μm in thickness. The sections were baked at 65°C for 1 h, processed through xylene dewaxing, rehydrated in alcohol, stained with hematoxylin, differentiated in hydrochloric acid-alcohol, stained with eosin, dehydrated again in alcohol, cleared in xylene, and ultimately mounted with neutral balsam. Staining outcomes for each group were examined using a Leica bright field microscope (DM 2500).

### 2.10 Statistical methods

Data analysis and graph plotting were performed using SPSS 22.0 statistical software and OriginPro 8.5, respectively. The study employed a repeated measures design to evaluate differences in test indicators across groups and measurement times, as well as to track within-group changes over time. Normality of the data was assessed using the Shapiro-Wilk test. Quantitative data were presented as mean ± standard deviation (M ± SD). For analyses of within-subject effects, results assuming sphericity were used when the data met this assumption. When the sphericity assumption was violated, within-subject effects were adjusted using the Greenhouse-Geisser correction. Multiple comparisons in the multivariate tests were conducted using the Bonferroni correction method. A significance threshold of P < 0.05 was established for statistical significance.

## 3 Results

### 3.1 Teriparatide enhances autonomous exploratory behavior and hindlimb motor function post-SCI in rats

**FIGURE 1:**
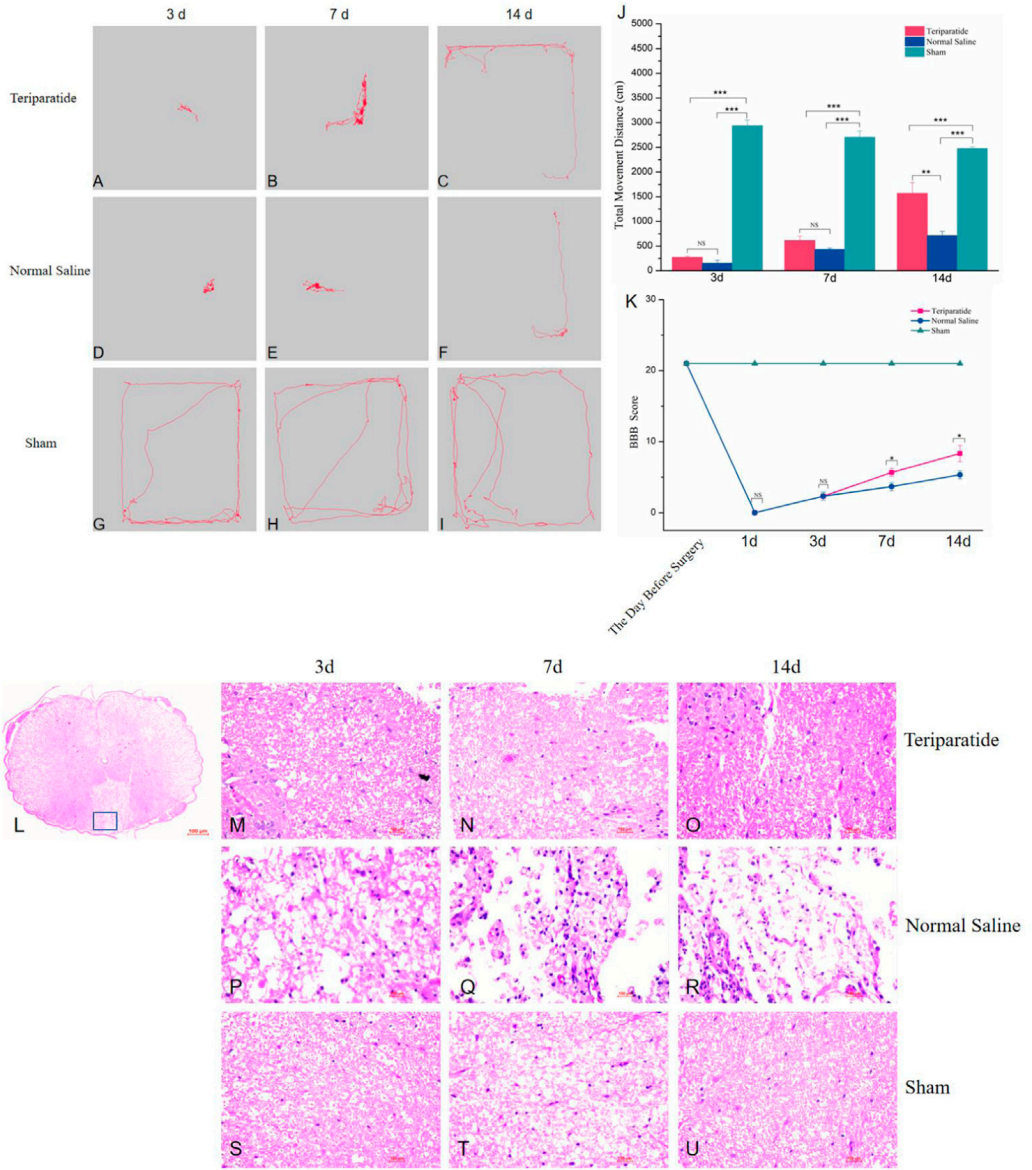
Illustrates the impact of teriparatide on the recovery of motor function in rats with SCI. **(A–C)** depict the locomotor trajectories during the open field test at 3, 7, and 14 days post-teriparatide administration, respectively. Correspondingly, **(D–F)** represent the trajectories for the saline-treated control group, while **(G–I)** display those for the sham-operated group at identical time points. **(J)** summarizes the statistical analysis of the total distance traveled in the open field test across the groups. **(K)** provides the BBB locomotor rating scale scores for each group, measured 1 day prior to surgery and at 1, 3, 7, and 14 days following treatment. **(L)** presents a comprehensive view of the spinal cord tissue stained with hematoxylin and eosin (H&E). **(M–O)** illustrate the H&E staining patterns at 3, 7, and 14 days post-treatment with teriparatide, respectively. Similarly, **(P–R)** demonstrate the H&E staining following 3, 7, and 14 days of saline treatment, respectively. Lastly, **(S–U)** display the H&E staining results for the Sham group at the corresponding 3, 7, and 14-day intervals. Statistical significance is denoted as follows: NS p > 0.05, *p < 0.05, **p = 0.001, ***p < 0.001.

To evaluate the potential of teriparatide in facilitating neural functional recovery following SCI, we performed open-field tests to measure the spontaneous exploratory behavior in rats. The teriparatide-treated group demonstrated enhanced exploratory behavior at 3, 7, and 14 days post-treatment compared to the control group. At 3 days post-treatment, the movement distance of the teriparatide group was comparable to the saline-treated group (teriparatide: 270.5 ± 21.15 cm vs. saline: 154.09 ± 59.16 cm; p = 0.341; Figures 1A, D, J), yet significantly less than the sham-operated group (2938.4 ± 117.64 cm; p < 0.0001; Figures 1G, J). At 7 days, both groups showed improvement in movement distance from day 3, but the difference between them was not statistically significant (teriparatide: 614.38 ± 90.72 cm vs. saline: 431.25 ± 27.95 cm; p = 0.139; Figures 1B, E, J), and both remained significantly lower than the sham group (2708.18 ± 122.78 cm; p < 0.0001; Figures 1H, J). At 14 days, continued improvement was observed, with the teriparatide group exhibiting a more pronounced increase in movement distance (teriparatide: 1567.23 ± 220.08 cm vs. saline: 715.88 ± 83.32 cm; p = 0.001; Figures 1C, F, J). The discrepancy in movement distance relative to the sham group also diminished (sham: 2477.4 ± 34.24 cm; Figures 1I, J).

Furthermore, the BBB scoring system was employed to assess hindlimb functional recovery. Prior to surgery, all rat groups scored 21 points on the BBB scale. On the first day post-treatment, both groups’ BBB scores dropped to 0 points (Figure 1K). By the third day, the scores remained comparable between groups (2.33 ± 0.58 for both, Figure 1K). On the seventh day, both groups exhibited improved BBB scores, with the teriparatide group outperforming the normal saline group (5.67 ± 0.58 vs. 3.67 ± 0.58, p = 0.013, Figure 1K). By the 14th day, further improvements were noted, with the teriparatide group showing a more significant enhancement in BBB scores (8.33 ± 1.15 vs. 5.33 ± 0.58, p = 0.015, Figure 1K). These findings indicate that teriparatide may enhance both autonomous exploratory behavior in novel environments and hindlimb motor function in rats with SCI.

To examine the spinal cord tissue architecture and cellular integrity following various interventions, rats from the three specified groups were euthanized at 3, 7, and 14 days post-treatment. Subsequently, the spinal cord specimens were fixed with paraformaldehyde, dehydrated, paraffin-embedded, and sectioned for histological analysis using hematoxylin and eosin (H&E) staining. Histological examination revealed that the control group, which received saline, presented with extensive infiltration of inflammatory cells and pronounced damage to the tissue structure. Conversely, the group treated with the teriparatide exhibited minimal inflammatory cell infiltration, with notably less tissue damage in comparison to the control group (Figures 1L–U).

### 3.2 Teriparatide enhances expression of antioxidant proteins HO-1 and SOD2 via upregulation of Nrf2

**FIGURE 2:**
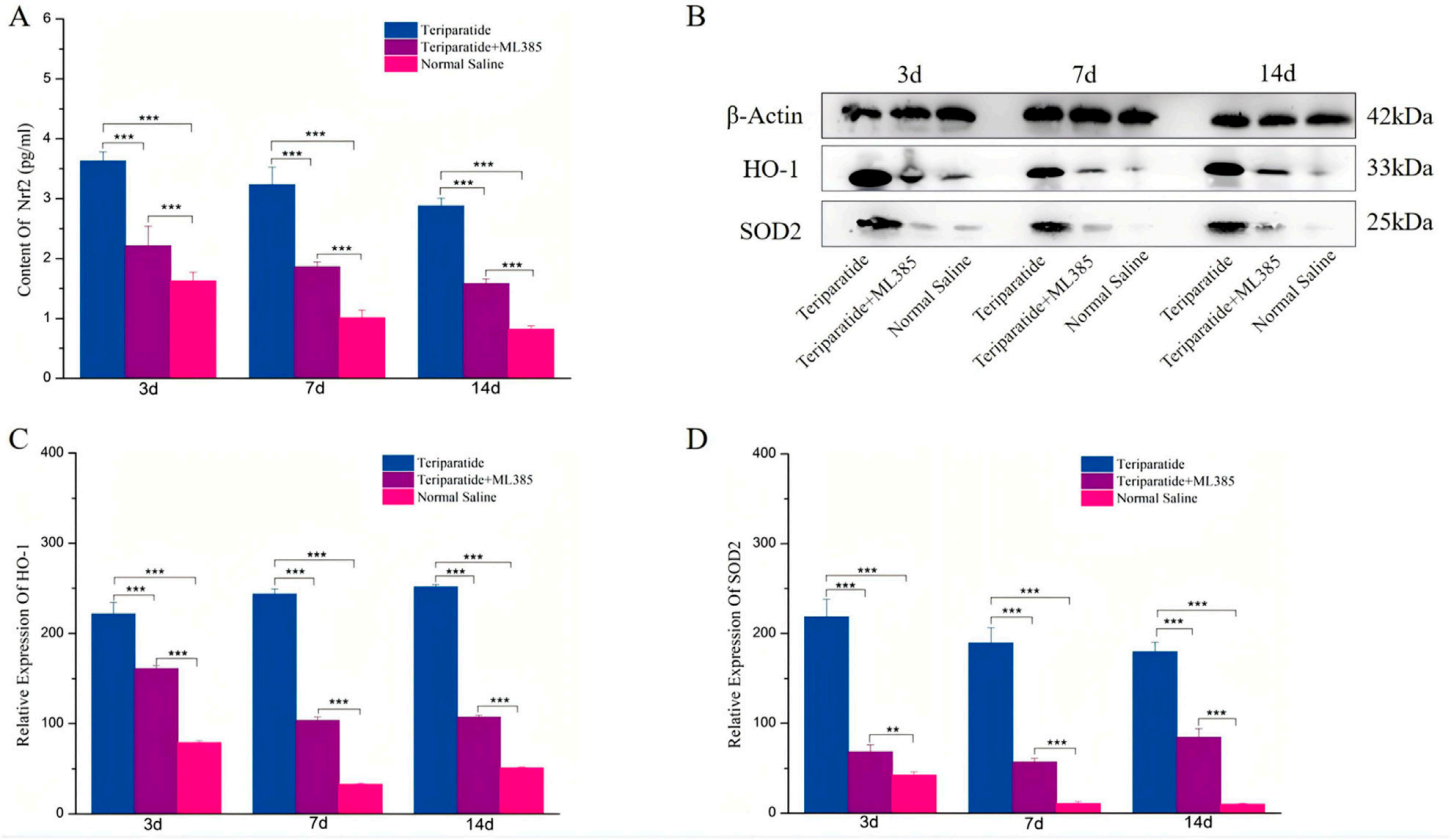
Illustrates the role of teriparatide in enhancing the expression of the antioxidant proteins HO-1 and SOD2 through the activation of Nrf2. Specifically, **(A)** presents the enzyme-linked immunosorbent assay (ELISA) quantification of Nrf2 levels in the teriparatide-treated group, the combination group (teriparatide + ML385), and the saline control group at intervals of 3, 7, and 14 days post-treatment. **(B)** displays the corresponding Western blot analysis for HO-1 and SOD2 across the same groups and time points. **(C, D)** detail the comparative expression levels of HO-1 and SOD2, respectively, following 3, 7, and 14 days treatment periods in each experimental group. Statistical significance is indicated by asterisks, with ** denoting p < 0.01 and *** denoting p < 0.001.

Our previous research demonstrated that teriparatide mitigates oxidative stress and ameliorates neurological symptoms in a rat model of ischemic cerebral infarction by activating the Nrf2/HO-1 signaling pathway (Xiong et al., 2022). To determine the role of this pathway in the therapeutic effects of teriparatide following SCI, we randomly assigned spinal cord-injured rats to one of three groups: a teriparatide-treated group, a group receiving teriparatide and the Nrf2 inhibitor ML385, and a control group administered normal saline. We quantified Nrf2 levels using an ELISA assay. At 3 days of treatment, Nrf2 levels in the teriparatide group were significantly elevated compared to the control group (3.68 ± 0.15 vs. 1.63 ± 0.14, p < 0.001; Figure 2A). Treatment with ML385 reduced Nrf2 levels (3.68 ± 0.15 vs. 2.21 ± 0.33, p < 0.001; Figure 2A), yet they remained higher than those in the control group (2.21 ± 0.33 vs. 1.63 ± 0.14, p < 0.001; Figure 2A). At 7 days, the teriparatide group maintained higher Nrf2 levels than both the teriparatide + ML385 and control groups (3.23 ± 0.29 vs. 1.86 ± 0.08 and 1.01 ± 0.12, respectively, p < 0.001; Figure 2A). This trend persisted at 14 days, with the teriparatide group showing higher Nrf2 levels than the teriparatide + ML385 and control groups (2.87 ± 0.13 vs. 1.58 ± 0.07 and 0.82 ± 0.06, respectively, p < 0.001; Figure 2A). These findings suggest that teriparatide enhances Nrf2 production in a rat model of SCI, potentially elucidating one mechanism by which it improves neurological outcomes.

To investigate the role of Teriparatide in the induction of downstream antioxidant stress proteins through the upregulation of Nrf2, we employed Western Blot analysis to measure the expression levels of heme oxygenase-1 HO-1 and SOD2 in each experimental groups. After 3 days of treatment, Western Blot data revealed a significant increase in HO-1 and SOD2 expression in the Teriparatide-treated group compared to both the Teriparatide + ML385 and the normal saline control groups (HO-1: Teriparatide vs. Teriparatide + ML385, 221.58 ± 12.8 vs. 161.21 ± 3.48, p < 0.001; Teriparatide vs. Normal Saline, 221.58 ± 12.8 vs. 79.01 ± 2.15, p < 0.001; SOD2: Teriparatide vs. Teriparatide + ML385, 218.32 ± 19.78 vs. 68.2 ± 7.84, p < 0.001; Teriparatide vs. Normal Saline, 218.32 ± 19.78 vs. 42.45 ± 3.5, p < 0.001; Figures 2B–D). Additionally, the Teriparatide + ML385 group exhibited higher levels of these proteins than the normal saline group, with significant differences (Figures 2B–D). At 7 days, the Teriparatide group continued to show significantly elevated levels of HO-1 and SOD2 compared to the Teriparatide + ML385 and normal saline groups (HO-1: Teriparatide vs. Teriparatide + ML385, 243.89 ± 5.39 vs. 103.51 ± 3.98, p < 0.001; Teriparatide vs. Normal Saline, 243.89 ± 5.39 vs. 32.72 ± 1.69, p < 0.001; SOD2: Teriparatide vs. Teriparatide + ML385, 189.48 ± 16.95 vs. 56.69 ± 4.13, p < 0.001; Teriparatide vs. Normal Saline, 189.48 ± 16.95 vs. 10.62 ± 2.68, p < 0.001; Figures 2B–D). The Teriparatide + ML385 group also maintained higher expression levels than the normal saline group, with statistically significant differences (Figures 2B–D). Consistent with earlier observations, at 14 days, the Teriparatide group demonstrated a significantly higher expression of HO-1 and SOD2 proteins relative to the Teriparatide + ML385 and normal saline groups (HO-1: Teriparatide vs. Teriparatide + ML385, 251.42 ± 2.72 vs. 107.19 ± 2.27, p < 0.001; Teriparatide vs. Normal Saline, 251.42 ± 2.72 vs. 51.77 ± 1.05, p < 0.001; SOD2: Teriparatide vs. Teriparatide + ML385, 179.66 ± 10.67 vs. 84.51 ± 9.5, p < 0.001; Teriparatide vs. Normal Saline, 179.66 ± 10.67 vs. 10.16 ± 1.15, p < 0.001; Figures 2B–D). Similarly, the Teriparatide + ML385 group’s expression of these proteins was also significantly greater than that in the normal saline group (Figures 2B–D). Collectively, these results indicate that Teriparatide can stimulate the expression of the antioxidant proteins HO-1 and SOD2 by enhancing Nrf2 production.

**FIGURE 3:**
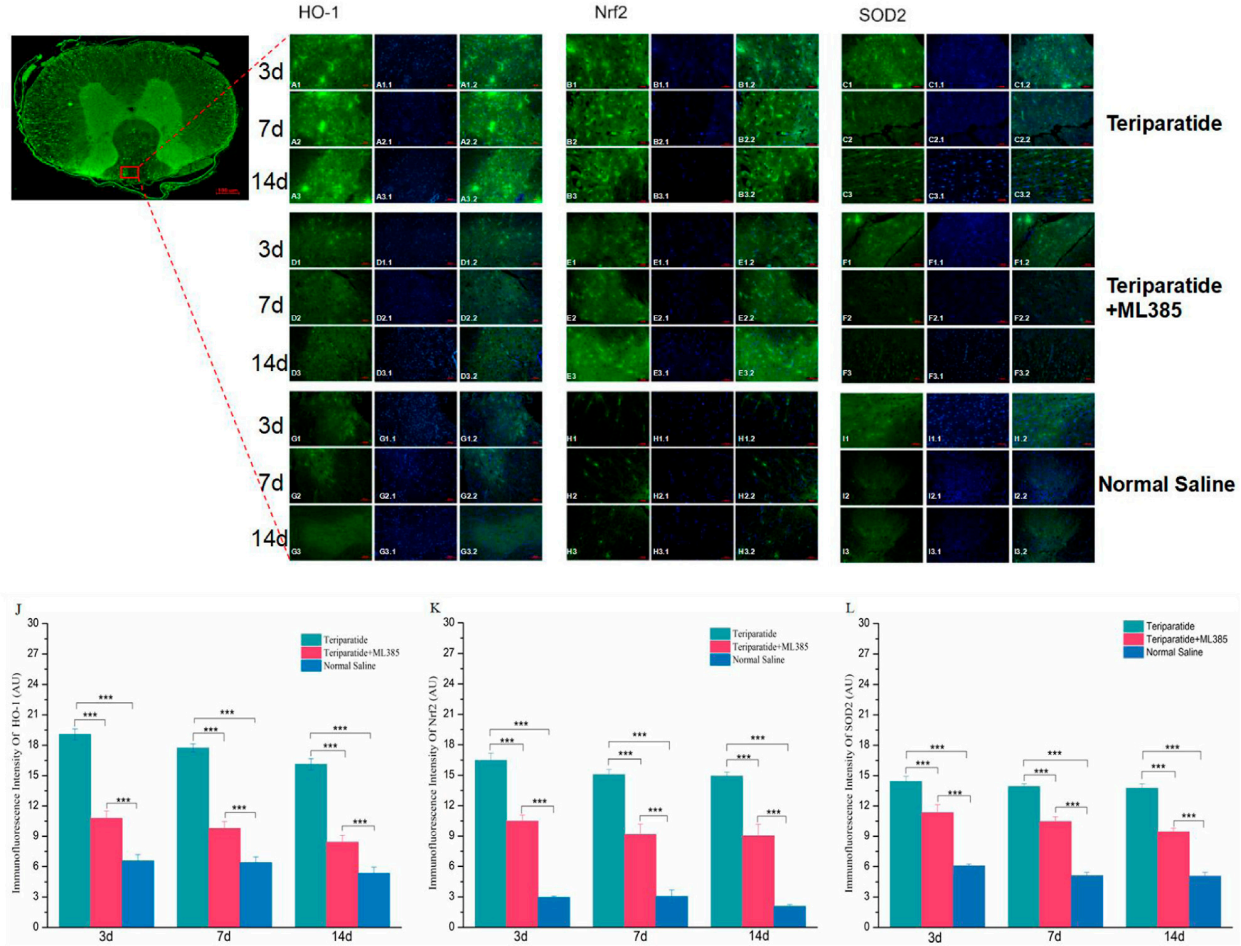
Teriparatide Induces Upregulation of Nrf2, HO-1, and SOD2 in Spinal Cord Tissues. In this figure comprises immunofluorescence staining of Nrf2, HO-1, and SOD2 in spinal cord tissues following teriparatide treatment. **(A1–A3, B1–B3, C1–C3)** depict the expression of HO-1, Nrf2 and SOD2, respectively, in the teriparatide-treated group at 3, 7, and 14 days post-treatment, with a magnification of ×20. Correspondingly, **(D1–D3, E1–E3, F1–F3)** illustrate the expression in the teriparatide + ML385 group, while **(G1–G3, H1–H3, I1–I3)** show the expression in the saline control group at identical time points and magnification. Quantitative analyses of the immunofluorescence intensity for HO-1,Nrf2 and SOD2 across the groups at 3, 7, and 14 days are presented in **(J–L)**, respectively. Statistically significant differences are indicated with asterisks (***p < 0.001).

To observe the expression of Nrf2, HO-1, and SOD2 in the spinal cord tissue of rats with SCI treated with teriparatide, we employed immunofluorescence techniques. The results showed that at 3 days of treatment, the immunofluorescence intensity of Nrf2, HO-1, and SOD2 in the teriparatide group was significantly higher than that in both the teriparatide + ML385 group and the normal saline group (**Nrf2:** Teriparatide vs. Teriparatide + ML385 16.47 ± 0.72 vs. 11.2 ± 0.6, Figures 3B1, D1, K, Teriparatide vs. Normal Saline 16.47 ± 0.72 vs. 2.96 ± 0.12; Figures 3B1, H1, K. **HO-1:** Teriparatide vs. Teriparatide + ML385 19.07 ± 0.54 vs. 10.78 ± 0.72; Figures 3A1, D1, J. Teriparatide vs. Normal Saline 19.07 ± 0.54 vs. 6.58 ± 0.64; Figures 3A1, G1, J. **SOD2:** Teriparatide vs. Teriparatide + ML385 14.41 ± 0.53 vs. 11.3 ± 0.798; Figures 3C1, F1, L, Teriparatide vs. Normal Saline 14.41 ± 0.53 vs. 6.08 ± 0.18; Figures 3C1, I1, L. p < 0.001). The teriparatide + ML385 group also exhibited higher immunofluorescence intensity for Nrf2, HO-1, and SOD2 compared to the normal saline group (**Nrf2:** Teriparatide + ML385 vs. Normal Saline 11.2 ± 0.6 vs. 2.96 ± 0.12, Figures 3E1, H1, 1. **HO-1:** Teriparatide + ML385 vs. Normal Saline 10.78 ± 0.72 vs. 6.57 ± 0.64; Figures 3D1, G1, J. **SOD2:** Teriparatide + ML385 vs. Normal Saline 11.33 ± 0.79 vs. 6.08 ± 0.18; Figures 3F1, I1, L. P < 0.001).

At 7 days of treatment, the immunofluorescence intensity of Nrf2, HO-1, and SOD2 in the teriparatide group was significantly elevated compared to the teriparatide + ML385 and normal saline groups (**Nrf2:** Teriparatide vs. Teriparatide + ML385 15.06 ± 0.52 vs. 9.15 ± 1.04, Figures 3B2, E2, K, Teriparatide vs. Normal Saline 15.06 ± 0.52 vs. 3.04 ± 0.662; Figures 3B2, H2, K. **HO-1:** Teriparatide vs. Teriparatide + ML385 17.73 ± 0.4 vs. 9.78 ± 0.68; Figures 3A2, D2, J. Teriparatide vs. Normal Saline 17.73 ± 0.4 vs. 6.39 ± 0.58; Figures 3A2, G2, J. **SOD2:** Teriparatide vs. Teriparatide + ML385 13.92 ± 0.27 vs. 10.46 ± 0.47; Figures 3C2, F2, L, Teriparatide vs. Normal Saline 13.92 ± 0.27 vs. 5.11 ± 0.33; Figures 3C2, I2, L p < 0.001). Additionally, the teriparatide + ML385 group’s immunofluorescence intensity for Nrf2, HO-1, and SOD2 was also higher than that of the normal saline group (Nrf2: Teriparatide + ML385 vs. Normal Saline 9.15 ± 1.04 vs. 3.04 ± 0.66, Figures 3E2, H2, K. **HO-1:** Teriparatide + ML385 vs. Normal Saline 9.78 ± 0.68 vs. 6.38 ± 0.58; Figures 3D2, G2, J. **SOD2:** Teriparatide + ML385 vs. Normal Saline 10.46 ± 0.47 vs. 5.11 ± 0.33; Figures 3F2, I2, L P < 0.001).

At 14 days of treatment, similar to the previous two time points, the immunofluorescence intensity of Nrf2, HO-1, and SOD2 in the teriparatide group was significantly higher than that in both the teriparatide + ML385 and normal saline groups (**Nrf2:** Teriparatide vs. Teriparatide + ML385 14.91 ± 0.4 vs. 9.03 ± 1.13, Figures 3B3, E3, K, Teriparatide vs. Normal Saline 14.91 ± 0.4 vs. 2.07 ± 0.18; Figures 3B3, H3, K. **HO-1:** Teriparatide vs. Teriparatide + ML385 16.1 ± 0.56 vs. 8.41 ± 0.69; Figures 3A3, D3, J. Teriparatide vs. Normal Saline 16.1 ± 0.56 vs. 5.33 ± 0.62; Figures 3A3, G3, J. **SOD2:** Teriparatide vs. Teriparatide + ML385 13.75 ± 0.43 vs. 9.43 ± 0.38; Figures 3C3, F3, L, Teriparatide vs. Normal Saline 13.75 ± 0.43 vs. 5.06 ± 0.38; Figures 3C3, I3, L p < 0.001), and the teriparatide + ML385 group’s immunofluorescence intensity for Nrf2, HO-1, and SOD2 was also higher than the normal saline group (**Nrf2:** Teriparatide + ML385 vs. Normal Saline 9.03 ± 1.13 vs. 2.07 ± 0.18, Figures 3E3, H3, K. **HO-1:** Teriparatide + ML385 vs. Normal Saline 8.41 ± 0.69 vs. 5.33 ± 0.62; Figures 3D3, G3, J. **SOD2:** Teriparatide + ML385 vs. Normal Saline 9.43 ± 0.38 vs. 5.06 ± 0.38; Figures 3F3, I3, L P < 0.001).

### 3.3 Teriparatide mitigates oxidative stress following SCI via the Nrf2/HO-1 pathway

**FIGURE 4:**
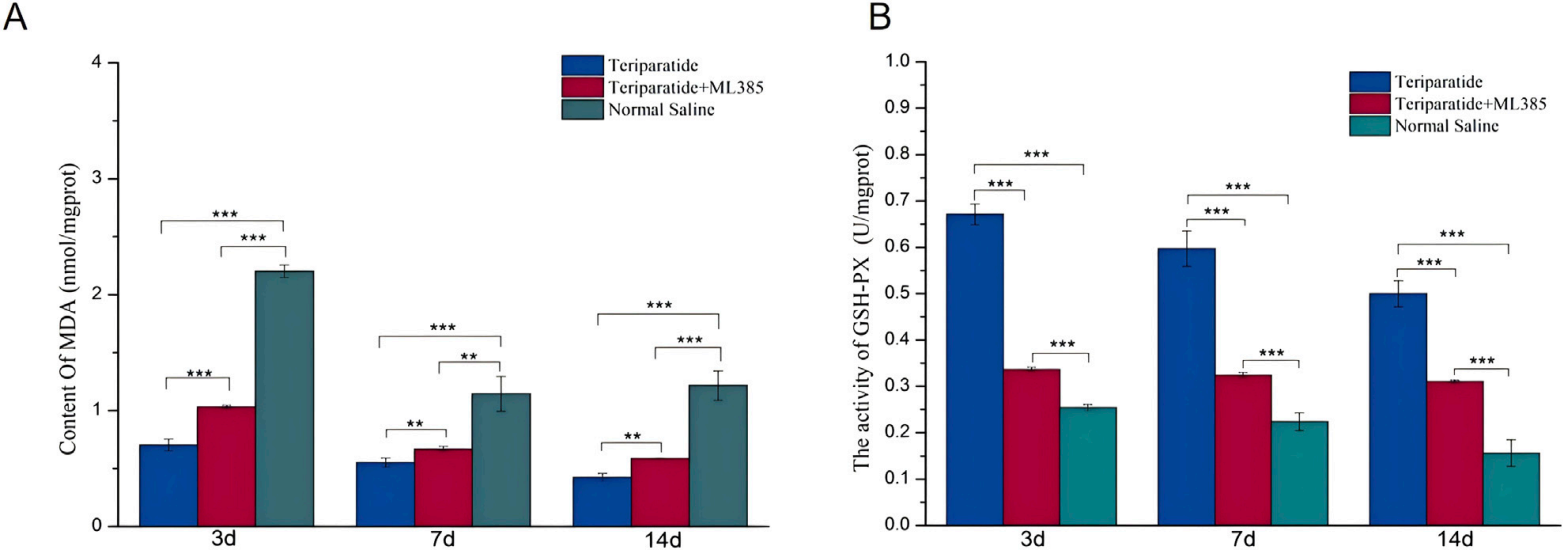
Illustrates the impact of teriparatide on oxidative stress in the context of SCI via the Nrf2/HO-1 signaling pathway. In **(A)**, the MDA levels, determined using an MDA assay kit, are compared across three groups: teriparatide-treated, teriparatide + ML385 (an Nrf2 inhibitor), and saline-treated controls at intervals of 3, 7, and 14 days post-treatment. **(B)** depicts the GSH-PX activity in the same groups over the same time periods, with statistical significance denoted by ** (p < 0.01) and *** (p < 0.001).

When SCI occurs, Nrf2 is activated, which prompts the upregulation of various antioxidant enzymes, including heme oxygenase-1 (HO-1) and superoxide dismutase 2 (SOD2). These enzymes play a pivotal role in neutralizing free radicals, thus reducing reactive oxygen species (ROS) production and mitigating cellular oxidative stress (Zhao et al., 2022; Jiang and He, 2022). Due to the transient nature of free radicals and the challenges associated with their direct assessment, quantifying their activity and end products, such as malondialdehyde (MDA), provides a more reliable measure post-SCI (Alizadeh et al., 2019). In our study, we randomly assigned SCI model rats to three groups: Teriparatide, Teriparatide + ML385 (an Nrf2 inhibitor), or Normal Saline. Using an MDA assay kit, we quantified the MDA levels in each group. At 3 days of treatment, the Teriparatide group exhibited a significant reduction in MDA levels compared to both the Teriparatide + ML385 and Normal Saline groups (0.7 ± 0.05 vs. 1.03 ± 0.02 and 2.2 ± 0.05 respectively; p < 0.001 for both comparisons, Figure 4A). The Teriparatide + ML385 group also showed a reduction in MDA levels compared to the Normal Saline group (1.03 ± 0.02 vs. 2.2 ± 0.05; p < 0.001, Figure 4A). At 7 days of treatment, this pattern persisted, with the Teriparatide group maintaining significantly lower MDA levels than the Teriparatide + ML385 and Normal Saline groups (0.55 ± 0.04 vs. 0.67 ± 0.02 and 1.15 ± 0.15 respectively; p = 0.002 and p < 0.001, Figure 4A). The Teriparatide + ML385 group again demonstrated lower MDA levels than the Normal Saline group (0.67 ± 0.02 vs. 1.15 ± 0.15; p = 0.005, Figure 4A). At 14 days of treatment, the trend continued, with the Teriparatide group showing the lowest MDA levels compared to the Teriparatide + ML385 and Normal Saline groups (0.42 ± 0.03 vs. 0.57 ± 0.01 and 1.22 ± 0.13 respectively; p < 0.001 for both comparisons, Figure 4A). The Teriparatide + ML385 group also maintained lower MDA levels than the Normal Saline group (0.57 ± 0.01 vs. 1.22 ± 0.13; p < 0.01, Figure 4A). These findings suggest that Teriparatide effectively reduces MDA production through the Nrf2/HO-1 signaling pathway, thereby attenuating oxidative stress after SCI.

Prior research has established that glutathione (GSH), an antioxidant, can mitigate neuronal oxidative damage by regulating the production and detoxification of free radicals (Wei et al., 2020). To evaluate the antioxidative capacity of teriparatide, we quantified the activity of glutathione peroxidase (GSH-PX) using an assay kit. Rats with induced SCI were randomly allocated into three groups: the teriparatide-treated group, the teriparatide plus ML385 group (where ML385 serves as an Nrf2 inhibitor), and the control group receiving normal saline. At 3 days of treatment, GSH-PX activity in the teriparatide-treated group was significantly higher than in both the teriparatide plus ML385 group and the normal saline group (teriparatide vs. teriparatide + ML385: 0.67 ± 0.02 vs. 0.34 ± 0.005, p < 0.001; teriparatide vs. normal saline: 0.67 ± 0.02 vs. 0.25 ± 0.007, p < 0.001; see Figure 4B). GSH-PX activity was also higher in the teriparatide plus ML385 group compared to the normal saline group (0.34 ± 0.005 vs. 0.25 ± 0.007, p < 0.001; Figure 4B). At 7 days, the teriparatide-treated group continued to exhibit enhanced GSH-PX activity relative to the other two groups (teriparatide vs. teriparatide + ML385: 0.6 ± 0.04 vs. 0.32 ± 0.006, p < 0.001; teriparatide vs. normal saline: 0.6 ± 0.04 vs. 0.22 ± 0.02, p < 0.001; Figure 4B). The teriparatide plus ML385 group also showed increased GSH-PX activity compared to the normal saline group (0.32 ± 0.006 vs. 0.22 ± 0.02, p < 0.001; Figure 4B), with significant differences observed. At 14 days, the pattern persisted, with the teriparatide-treated group demonstrating superior GSH-PX activity over the teriparatide plus ML385 group and the normal saline group (teriparatide vs. teriparatide + ML385: 0.5 ± 0.03 vs. 0.31 ± 0.004, p < 0.001; teriparatide vs. normal saline: 0.5 ± 0.03 vs. 0.16 ± 0.03, p < 0.001; Figure 4B). Furthermore, the teriparatide plus ML385 group maintained higher GSH-PX activity than the normal saline group (0.31 ± 0.004 vs. 0.16 ± 0.03, p < 0.01; Figure 4B). Collectively, these findings indicate that teriparatide may exert antioxidative effects and contribute to the mitigation of oxidative stress following SCI.

## 4 Discussion

Over the past 2 decades, the global incidence of SCI has risen markedly, positioning it as a particularly severe condition within the hierarchy of global disease burden (Global, 2023). Enhancing SCI prognosis is imperative for reducing societal impacts, and the pursuit of novel therapeutic strategies is of paramount importance. In this study, we present the first investigation, to our knowledge, of teriparatide’s therapeutic effects in a rat model of SCI. Our findings indicate that teriparatide treatment not only ameliorates motor and exploratory functions in SCI-afflicted rats but also augments glutathione peroxidase (GSH-PX) activity post-injury and diminishes the levels of the oxidative stress biomarker malondialdehyde (MDA). Through a comprehensive analysis of the underlying mechanisms, we discovered that teriparatide treatment elevates Nrf2 production and enhances the expression of the downstream antioxidant proteins HO-1 and SOD2. These findings imply that teriparatide may favorably influence SCI prognosis by mitigating oxidative stress.

Teriparatide, or recombinant human parathyroid hormone, is recognized for its robust osteogenic properties and is extensively utilized in treating postmenopausal osteoporosis (Rendina-Ruedy and Rosen, 2022). In 1994, Vaziri first identified a link between parathyroid hormone and SCI, observing a notable decline in parathyroid hormone (PTH) levels in a cohort of 40 SCI patients (Vaziri et al., 1994). More recent research suggests that individuals with complete paraplegia due to SCI exhibit a greater decrease in PTH levels than those with incomplete paraplegia, suggesting a possible association between the severity of neural damage and PTH reduction (Mechanick et al., 1997; Ouyang et al., 2021). Despite the potential therapeutic benefits, there remains a paucity of research, both domestically and internationally, on the application of parathyroid hormone and its analogs in SCI treatment. Our research involving the administration of teriparatide to rats with SCI yielded promising results. In the open field test, the teriparatide-treated group demonstrated a significantly increased motor distance compared to the control group. Moreover, the SCI score, as assessed by the Basso, Beattie, and Bresnahan (BBB) scale, also indicated substantial improvement. These findings suggest that teriparatide may ameliorate symptoms of neural damage in SCI, aligning with the conclusions of prior studies.

During SCI, ischemia and hypoxia precipitate an overproduction of free radicals. These unstable molecules react with proteins, lipids, DNA, and other cellular components, inflicting toxic effects and causing irreversible damage to neuronal cells (Yu et al., 2023). Accumulating evidence indicates that mitigating oxidative product formation post-SCI can substantially protect neurons from oxidative stress, thereby aiding in neuronal repair and axonal regeneration (Rosales-Antequera et al., 2022; Chen et al., 2021). Research has revealed that teriparatide, known primarily for its regulatory effect on bone metabolism, also has the capacity to modulate oxidative stress. In patients with osteoporosis, therapeutic doses of teriparatide have been shown to decrease oxidative product formation in bone cells, effectively attenuating oxidative stress responses (Gambardella et al., 2018; Guo et al., 2022). Our prior studies demonstrated that teriparatide activates the Nrf2 pathway in cerebral ischemia-reperfusion injury, which promotes the expression of the antioxidant proteins HO-1 and SOD2. This activation yields an antioxidative effect, significantly reducing damage to the central nervous system and enhancing the restoration of neural functions (Xiong et al., 2022). To determine whether teriparatide’s amelioration of neural symptoms following SCI is attributable to its antioxidative properties, we initially assessed the activity of glutathione peroxidase (GSH-PX). Our findings indicated that GSH-PX activity in the teriparatide-treated group was markedly higher than in the control group. Furthermore, we observed a significant reduction in the levels of the oxidative stress biomarker malondialdehyde (MDA) in the teriparatide group compared to controls, corroborating the antioxidative impact of teriparatide.

Research has demonstrated that the Nrf2/HO-1 signaling pathway is implicated in the secondary cascade of reactions following SCI and exerts a substantial protective influence on the pathological progression of SCI (Guo et al., 2022). Nrf2 is a crucial transcription factor involved in the cellular antioxidant response (Scuderi et al., 2020). Under normal conditions, Nrf2 remains inactive in the cytoplasm through its association with Keap1 (Guo et al., 2022; Cano et al., 2021). Upon SCI, Nrf2 activation occurs, leading to the dissociation of the Nrf2-Keap1 complex, phosphorylation of Nrf2, and its subsequent translocation to the nucleus. This process upregulates the expression of various antioxidant enzyme genes, including HO-1, NQO1, and superoxide dismutase (SOD), which directly or indirectly mitigate free radical damage, thereby attenuating the generation of reactive oxygen species (ROS) and inhibiting oxidative stress in cells (Zhao et al., 2022; Jiang and He, 2022). Heme oxygenase-1 (HO-1) serves as a stress-induced enzyme and plays a pivotal role in maintaining cellular homeostasis. It catalyzes the conversion of heme into biliverdin, which is further converted to bilirubin, providing a potent antioxidant effect (Consoli et al., 2021; Lianos and Detsika, 2023; Hirvonen et al., 2022). Because oxidative stress is a principal outcome of SCI, and both Nrf2 and HO-1 contribute to the mitigation of this stress, enhancing the expression of these proteins has been identified as a therapeutic strategy to improve SCI outcomes. Given the antioxidative properties of teriparatide, we hypothesized that its therapeutic effects might be mediated through the activation of the Nrf2/HO-1 pathway. We initiated our study by quantifying Nrf2 levels using ELISA and observed a significant elevation following teriparatide treatment. Furthermore, we assessed the expression of the downstream antioxidant proteins HO-1 and SOD2 through Western blot analysis, which confirmed that teriparatide treatment enhances the production of these proteins. In subsequent experiments, we employed a combination of teriparatide and the Nrf2 inhibitor ML385 in a rat SCI model. The outcomes showed that ML385 attenuated the upregulation of Nrf2, HO-1, and SOD2 induced by teriparatide. These results support the hypothesis that teriparatide ameliorates oxidative stress through the activation of the Nrf2/HO-1 signaling pathway, thereby facilitating the synthesis of downstream antioxidant proteins.

This study is subject to certain limitations. Our findings indicate that in the ML385 group, the activities of Nrf2, HO-1, SOD2, and GSH-PX were reduced compared to the teriparatide group, yet elevated relative to the saline control group. These results suggest that teriparatide may enhance the synthesis of downstream antioxidant proteins through the activation of the Nrf2/HO-1 signaling pathway, thereby manifesting its antioxidative effects. However, existing literature posits that the FoxO and NF-κB pathways may also modulate the activities of SOD2 and GSH-PX, contributing to oxidative stress regulation (Eijkelenboom and Burgering, 2013; Morgan and Liu, 2011). The exact relationship between teriparatide’s regulation of oxidative stress and these pathways remains to be elucidated. Future work will involve the use of transcriptomics to identify additional pathways that teriparatide may influence.

## 5 Conclusion

Teriparatide, widely utilized for osteoporosis management, has demonstrated neuroprotective properties in rodent models of SCI. It appears to induce the generation of antioxidant proteins through the activation of the Nrf2/HO-1 signaling pathway, diminish the production of the oxidative stress MDA, augment the activity of GSH-PX, and mitigate oxidative stress post-injury, thereby enhancing recovery prospects. These findings lay a scientific groundwork for future clinical trials to explore teriparatide’s therapeutic potential in treating spinal cord injuries. Moreover, the study introduces a novel pharmacological approach for SCI management.

## Data Availability

The raw data supporting the conclusions of this article will be made available by the authors, without undue reservation.
