# Supplementary figures and images for: Teriparatide mitigates oxidative stress following spinal cord injury and enhances neurological recovery via the Nrf2/HO-1 signaling pathway
Source: Front Pharmacol. 2025 Mar 19;16:1538857. doi: 10.3389/fphar.2025.1538857 (PMC11962027; doi:10.3389/fphar.2025.1538857)

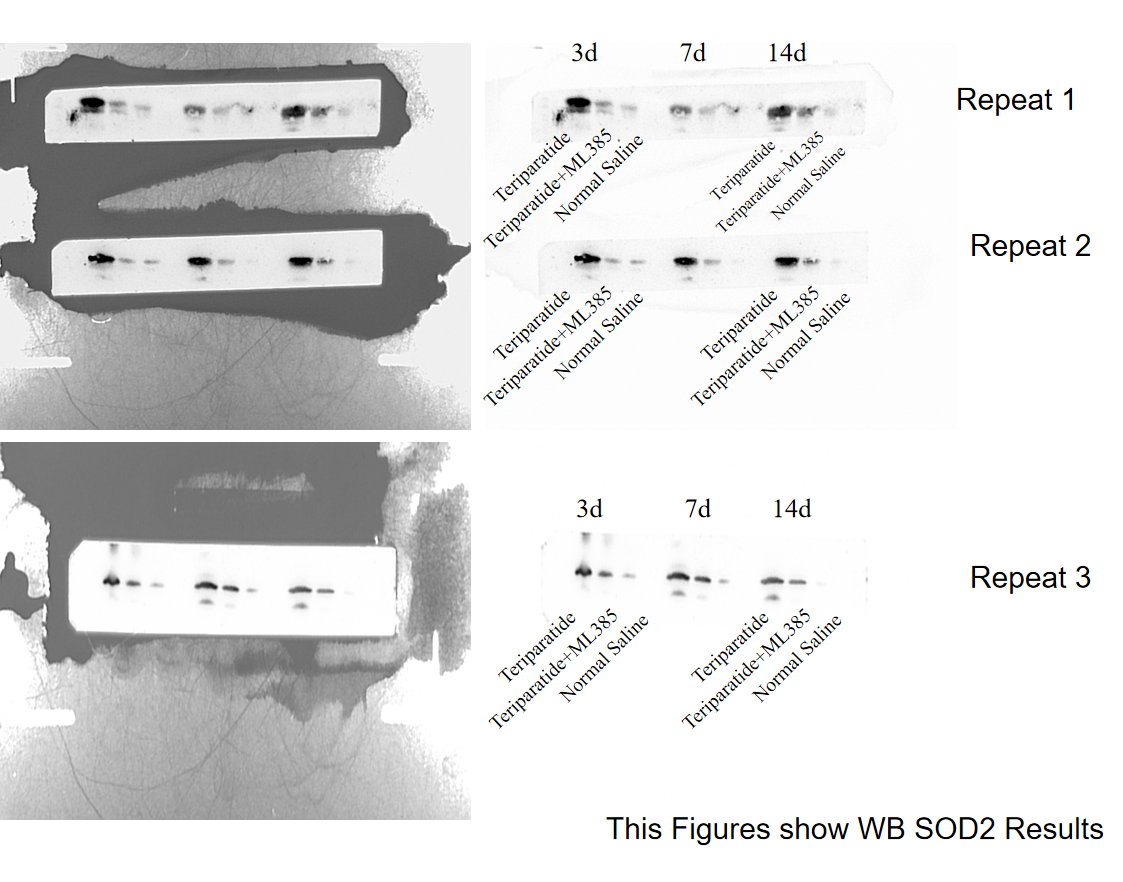

Supplement: Supplementary file 1 [file Image2.PNG]

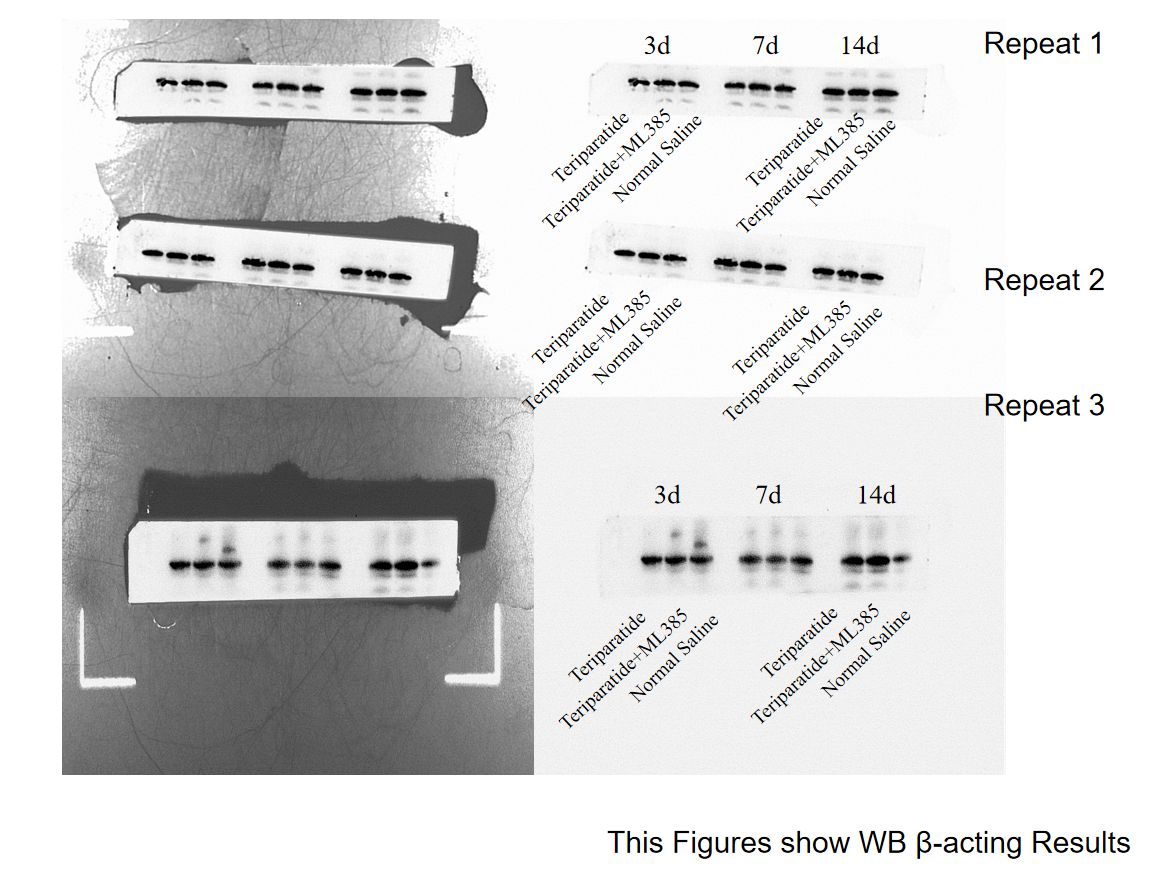

Supplement: Supplementary file 2 [file Image1.PNG]

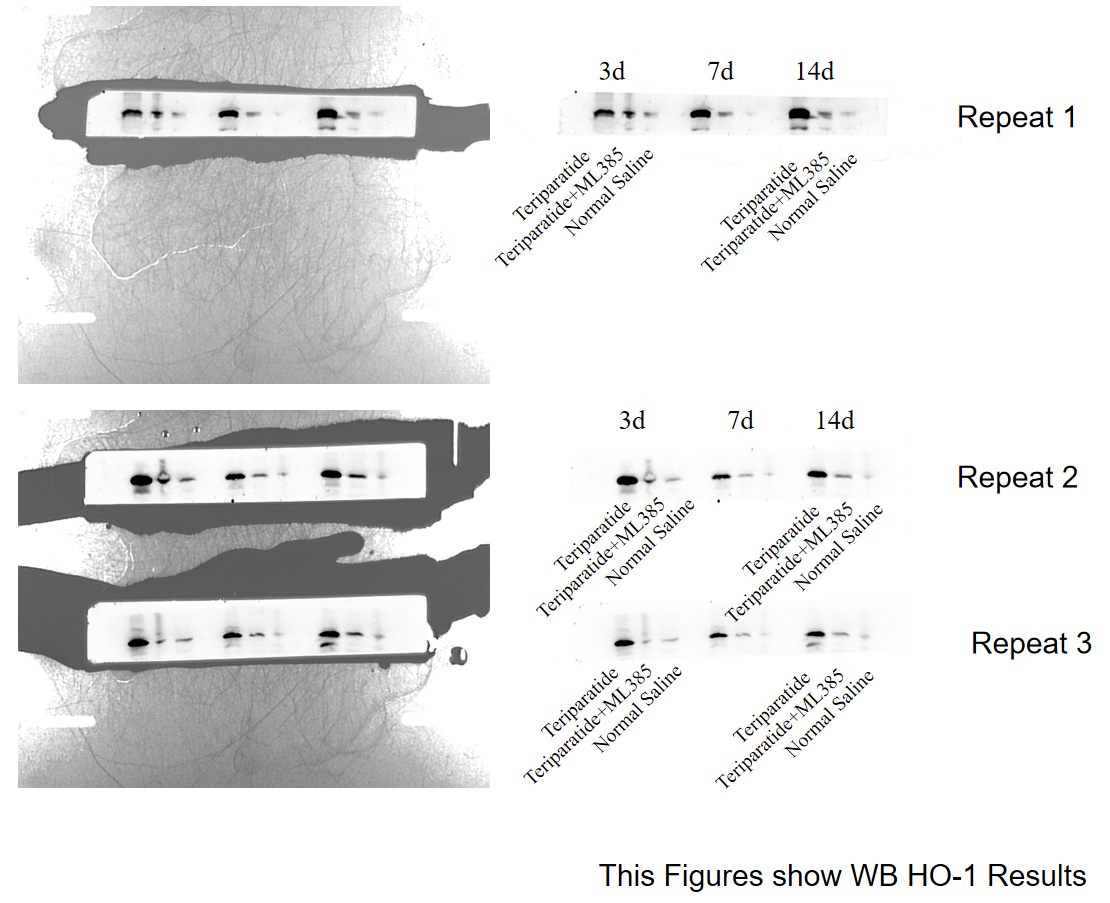

Supplement: Supplementary file 3 [file Image3.PNG]
